# Bilateral Primary testicular diffuse large B-CELL lymphoma

**DOI:** 10.1016/j.eucr.2021.101733

**Published:** 2021-05-28

**Authors:** Francesco Trama, Ester Illiano, Achille Aveta, Savio Domenico Pandolfo, Giancarlo Bertuzzi, Elisabetta Costantini

**Affiliations:** a Andrology and Urogynecology Clinic, Santa Maria Terni Hospital, University of Perugia, Italy; b Department of General and Specialized Surgeries, Renal Transplantation, Nephrology, Intensive Care and Pain Management, University of Federico II, Naples, Italy

**Keywords:** Primary testicular lymphoma, Non-hodgkin lymphoma, Bilateral testicular cancer, Bilateral orchiectomy

## Abstract

Bilateral Primary testicular lymphoma (PTL) is a rare and aggressive non-Hodgkin extranodal lymphoma. Despite this low overall incidence, it is the most common testicular cancer in the elderly. PTL is characterized by spreading to non-contiguous extranodal sites (especially in SNC), high recurrence rate and poor prognosis. We report a case of a 55-year old man with advanced bilateral PTL without specific symptoms who underwent a combined multimodal approach.

## Introduction

Primary testicular lymphoma (PTL) is an unusual and aggressive non-Hodgkin extranodal lymphoma with an annual incidence of 0.26 cases on 100,000 men. Although it represents only 3–9% of testicular cancers, it is the most common testicular tumor in men aged over 60. PTL also represents 1–2% of all non-Hodgkin lymphomas.^1^ The most common histotype of the PTL is Diffuse large B cell lymphoma (DLBCL). PTL is also considered the most common bilateral cancer of the testis, with an occurrence of bilateral metachronous testicular association of 35% and synchronous testicular involvement of 3%. In the majority of cases (50–60%) PTL appears with local involvement with testicular mass or swelling, mono or bilateral (stage I-E, II-E). Rarely it appears with extra-nodal and distant metastases (stage III-IV) and in those patients we can also find systematic symptoms such as fever, weight loss and night sweats (symptoms B).^1^

## Case report

A 55-year old man showed up in our clinic with bilateral increased testicular volume, pain, hard “woody” consistence and thickening of the scrotum. He has been treated over the last year for suspected bilateral orchiepididymitis with antibiotic and steroid therapy. The patient was not seen by a specialist due to the Coronoravirus pandemic. In fact, the patient was afraid to go to the urology clinic. He denied fever, weight loss and history of underscended testicular and scrotal surgery.

**Fig. 1:**
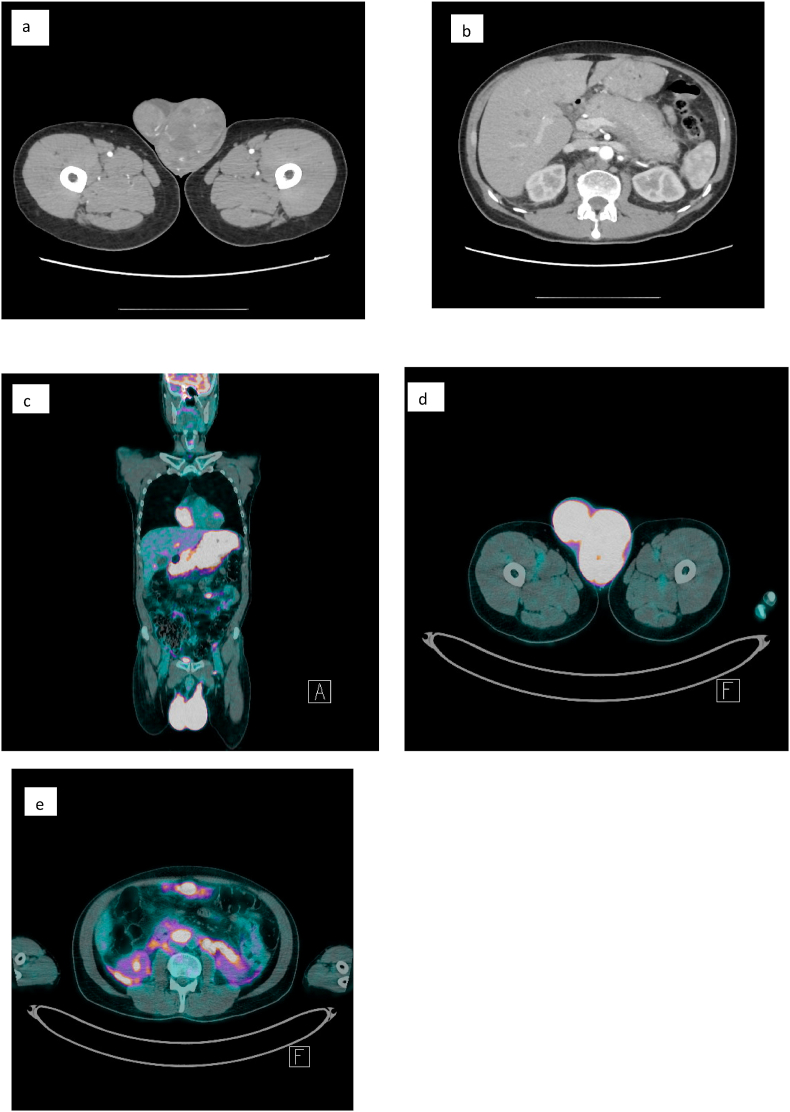
**(A**–**b) CT abdomen - pelvis with and without contrast**. Numerous supracentimetric lymphadenopathies were evident in the abdominal and At the level of the scrotum, markedly increased didymes bilaterally were highlighted with a strongly inhomogeneous appearance and with hypodense gaps in the context; the left didymus was more enlarged and more inhomogeneous and shows axial diameters of 92 × 50 mm. **(c**–**e) 18F- FDG PET:** The presence of pathological hyperaccumulation of the radiopharmaceutical Was present in all body districts examined.

Ultrasonography revealed bilateral testicular enlargement (76 mm right testis, 100 mm left testis) with multiple lumbo-aortic lymphadenopathies, the largest measuring 38 mm. Computed tomography (CT) scan showed: round formation of cystic appearance located in the pancreatic head, thickened stomach wall, bilateral testicular enlargement and multiple enlarged lymph nodes (inferior paratracheal, retrocrural, interaortocaval, perigrastric, and left lumbo-aortic) (Fig. 1 a – b).

An upper gastrointestinal endoscopy was carried out which indicated the presence of hyperemia in the gastric fundus and gastric body and hypertrophic gastric rugae. Subsequent biopsy revealed Germinal Center B-Cell like (GCB) diffuse large B cell lymphoma (DLBCL) of the gastric body.

Afterwards, Positron emission tomography (PET) scanning demonstrated a conspicuous hypermetabolic lesion in different sites (right frontal sinus, maxillary sinuses, stomach, duodenum, pancreas, spinal canal from D9 to D10, right scapula, both testicles and multiple thoracic and abdominal lymph nodes) (Fig. 1 c - e).

In laboratory, Alpha-Fetoprotein (AFP) and Beta Human chorionic gonadotropin (BetaHCG) were within the normal limit, while LDH levels were elevated (676 UI/L). Serum antigens for immuno-deficiency virus (HIV) and serum antibodies against HIV were negative.

**Fig. 2:**
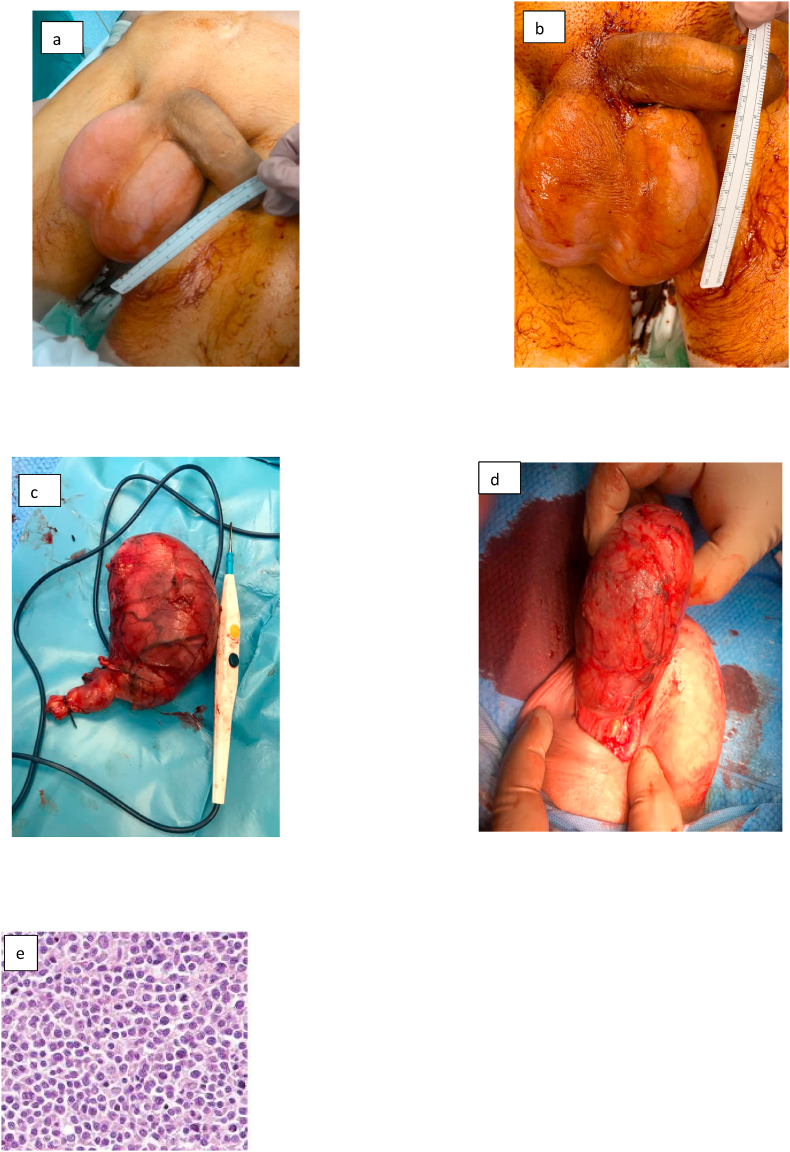
**(A**–**b)** perioperative specimens. **(c**–**e)** Intraoperative surgical specimens.

According to the presented symptoms, laboratory examinations and a bone marrow cell examination, the patient underwent bilateral radical orchiectomy, in agreement with radiotherapists, haematologists without surgery complications (Fig. 2a–d).

Orchiectomy Histopathology specimens revealed diffuse large B-cell lymphoma (DLBCL), in both testicles without c-Myc, BCL2 and BCL6 gene rearrangements (Fig. 2 e).

At immunohistochemistry analysis the neoplastic cells were CD20, Bcl6 and bcl2 positive; CD23 and CD5 negative. Ki67 positivity was above 95% in the areas with good fixing.

Following surgery, the patient received.

6 cycles of chemotherapy with the association of Cyclophosphamide, Doxorubicin, Vincristine and Prednisolone associated with immunotherapy with anti CD 20 antibodies. He received the treatment in day hospital without developing serious adverse events.

**Fig. 3:**
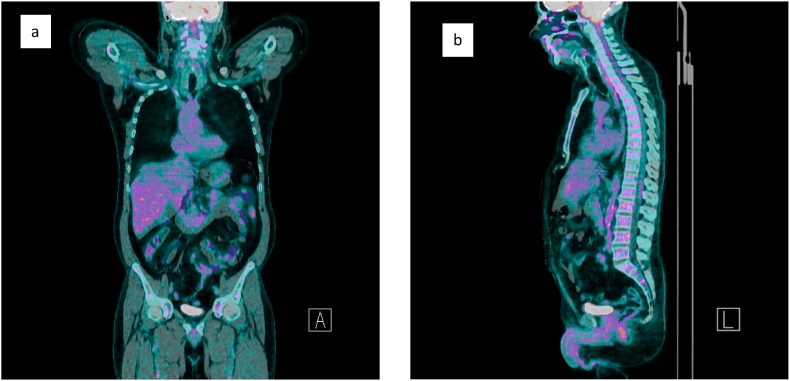
**18F- FDG PET after surgey and chemotherapy:** there is complete disappearance of the hyperfixations previously reported, demonstrating the complete response to the therapy in progress.

4 months after chemotherapy was completed, the PET scan revealed complete disappearance of the hypercaptations previously reported (Fig. 3 a – b). Subsequently, the patient was placed on the waiting list to perform bone marrow transplantation in order to consolidate the result achieved. Written consent was obtained from the patient and their relatives for publication of the study.

## Discussion

PTL (Primary Tumor Lymphoma) is the most common type of testicular tumor in men over 60, accounting for 1–2% of all non-Hodgkin lymphoma (NHL).^2^ In men under 60 bilateral testicular involvement and secondary testicular lymphoma are more common.^2^

The most common histological subtype in patients with PTL is the diffuse large B-cell lymphoma (DLBCL), representing about 80%–90% of testicular lymphoma.^2^ The prognosis for DLBCL is poor, and is negatively related to advanced stage, older age, B symptoms and high International Prognostic Index (IPI) score. Also elevated lactate dehydrogenase has been correlated with tumor aggressiveness, whereas Beta HCG and serum alpha-fetoprotein (AFP) are rarely elevated.^3^

Ann-Arbor staging is the staging system for PTL: stage-IE disease consists of the mono or bilateral involvement of the testes (50%–60% of all patients); stage-IIE disease represents the mono or bilateral testicular involvement with involvement of loco-regional (retroperitoneal and/or iliac) lymph nodes (20–30% of all patients); stage III-IV disease consists of the mono or bilateral testicular involvement with involvement of distant lymph nodes and/or extranodal sites.^1^

Advanced stages (stage III-IV) are very rare and are usually characterized by systemic B symptoms such as fever, night sweats and weight loss, in about 25–41% of patients.^2^

Currently due to low incidence and high aggressiveness, at the moment of diagnosis a multimodal treatment approach involving surgery, anthracycline-based combination chemotherapy (CHOP), prophylactic intrathecal chemotherapy and cranial/scrotal irradiation should be proposed to the patient. Despite there are no standard treatments, orchidectomy represents the initial treatment for all stage patients. In fact, it provides histological simples for diagnosis which is mandatory for the management of patients. In addition the chemotherapy would be vain in the testicle because of the blood testicular barrier that makes it a sanctuary site. Moreover the resistance to chemotherapy is also given by testicular tumor cells that can express high levels of drug-resistant proteins, such as *P*-glycoprotein (PGP) and breast cancer drug-resistant protein (BCRP).^4^ Adjuvant chemotherapy should be considered also in stage IE and IIE. The majority of patients treated with orchidectomy alone relapse within the first two years in various extranodal-sites.^5^ For advanced stages (IIIE, IVE) the standard treatment always includes systemic chemotherapy (Cyclophosphamide, Doxorubicin, Vincristine, Prendisolone), with irradiation reserved for symptomatic and Bulky localized deposits.

## Conclusion

Primary Testicular Lymphoma, although rare, represents the most pervasive kind of testicular tumor in men over 60. The similarity of symptoms with orichiepididymitis often delays the diagnosis. A multidisciplinary team, including urologists, medical and radiation oncologists, is required for treatment because standardized therapy has not yet been established.

## References

[1] Zucca E., Conconi A., Mughal T.I. (2003 Jan). Patterns of outcome and prognostic factors in primary large-cell lymphoma of the testis in a survey by the International Extranodal Lymphoma Study Group. J Clin Oncol.

[2] Swerdlow S.H., Campo E., Harris N.L. (2008). WHO Classification of Tumours of Haematopoietic and Lymphoid Tissues.

[3] Moller M.B., d'Amore F., Christensen B.E. (1994). Testicular lymphoma: a population-based study of incidence, clinicopathological correlations and prognosis. The Danish Lymphoma Study Group, LYFO. Eur J Canc.

[4] Bart J., Hollema H., Groen H.J. (2004 Sep). The distribution of drug-efflux pumps, P-gp, BCRP, MRP1 and MRP2, in the normal blood-testis barrier and in primary testicular tumours. Eur J Canc.

[5] Kondo T., Wada H., Yata K. (2002 Jun). [Seven patients with stage I and II primary testicular lymphoma]. Rinsho Ketsueki.

